# Integrated Approach Reveals Fermented *Moringa oleifera* Leaves Extracts’ Impact on Mouse Sleep

**DOI:** 10.3390/foods14172952

**Published:** 2025-08-25

**Authors:** Si Huang, Kuan Wu, Yuwei Guo, Hongyu Mu, Jun Sheng, Yang Tian, Jia Liu, Cunchao Zhao

**Affiliations:** 1 College of Food Science and Technology, Yunnan Agricultural University, Kunming 650201, China; 18870918872@163.com (S.H.); s1647025254@163.com (K.W.); 13874181211@163.com (Y.G.); muhongyu0710@163.com (H.M.); shengjunpuer@163.com (J.S.); tianyang1208@163.com (Y.T.); 2 College of Food, Nanchang University, Nanchang 330031, China; 3 Engineering Research Center of Development and Utilization of Food and Drug Homologous Resources Ministry of Education, Yunnan Agricultural University, Kunming 650201, China; 4 Yunnan Key Laboratory of Precision Nutrition and Personalized Food Manufacturing Ministry of Education, Yunnan Agricultural University, Kunming 650201, China; 5 China Yunnan Research Center for Advanced Tea Processing, Pu’er University, Pu’er 665000, China; 6 Yunnan Plateau Characteristic Agricultural Industry Research Institute, Yunnan Agricultural University, Kunming 650201, China; 7 College of Science, Yunnan Agricultural University, Kunming 650201, China; 8 Key Laboratory of Pu’er Tea Science, Ministry of Education, Yunnan Agricultural University, Kunming 650201, China

**Keywords:** *Moringa oleifera* leaves, sleep aid, metabolomics, network pharmacology, molecular docking

## Abstract

Sleep disturbances are linked to metabolic and neurological dysregulation. *Moringa oleifera* leaves, rich in bioactive compounds, may improve sleep via gut–brain axis modulation. This study investigated the sleep-enhancing effects of fermented *Moringa oleifera* leaf extract (FM) in mice using metabolomics, gut microbiota analysis, network pharmacology, and molecular docking. A 1:1 combination of Lactobacillus plantarum GDMCC 1.2685 and *L. swissii* GDMCC 1.791 optimally fermented FM, increasing GABA by 1.67-fold and total amino acids to 46,058.20 ± 845.53 μg/g. FM shortened sleep latency, increased sleep duration, and elevated brain GABA while reducing glutamate (Glu) and Glu/GABA ratios. Hypothalamic metabolomics identified seven sleep-related metabolites, implicating glycerophospholipid, tryptophan, and purine metabolism pathways. FM also reduced *Mycobacterium anisopliae* (a gut bacterium associated with insomnia) and increased the Firmicutes/Bacteroidetes ratio. Network pharmacology revealed that FM’s effects were mediated via GABA, Glu, and serotonin (5-HT) pathways. These findings demonstrate that FM improves sleep by modulating hypothalamic neurotransmitters and gut microbiota, exerting sedative-hypnotic effects through amino acid, purine, and energy metabolism.

## 1. Introduction

Sleep, as an important physiological regulatory process in the human body, has a profound impact on cognitive function, immune homeostasis and mood regulation through neuroendocrine regulatory networks [1]. Clinical studies have confirmed that sleep quality is significantly and positively correlated with hippocampus-dependent memory consolidation, prefrontal executive function and amygdala emotion regulation, while sleep deprivation can lead to decreased brain-derived neurotrophic factor (BDNF) levels and impaired synaptic plasticity in the prefrontal cortex [2]. At the immune level, sleep deprivation triggers the abnormal elevation of pro-inflammatory cytokines and the suppression of natural killer cell activity, which increases the risk of infection and the incidence of chronic inflammatory diseases [3]. Notably, chronic insomnia is present in approximately 30% of adults worldwide, and this persistent sleep disorder exhibits a dose–effect relationship with an increased risk of obesity, type 2 diabetes, and cardiovascular disease, with mechanisms that may involve dysregulation of the leptin/hunger hormone axis, sympathetic hyperactivity, and dysfunction of the hypothalamic–pituitary–adrenal (HPA) axis [4]. Although benzodiazepine receptor agonists (BZRAs) and novel non-benzodiazepine drugs are still the first-line clinical treatment options, the increasing problems of γ-aminobutyric acid (GABA) receptor desensitization, cognitive impairment, and withdrawal insomnia resulting from long-term use have prompted researchers to turn their attention to the development of natural products [5].

*Moringa oleifera*, a tropical plant in the family Moringaceae, is rich in multidimensional bioactive components in its leaves, including flavonoids with neuromodulatory effects, γ-aminobutyric acid that promotes GABA ergic neurotransmission, and dietary fibers that modulate the intestinal flora [6]. In vitro experiments have shown that *Moringa oleifera* leaves can significantly prolong the duration of sleep and shorten the sleep latency induced by sodium pentobarbital in mice by activating the α1 subunit of the GABA_A receptor and up-regulating the expression of glutamic acid decarboxylase [7]. More importantly, its unique isothiocyanate compounds can modulate the 5-hydroxytryptamine/dopaminergic pathway by inhibiting monoamine oxidase activity, which is a unique advantage for improving anxiety-related insomnia [8]. In addition, the antioxidant properties of *Moringa oleifera* can effectively scavenge the overproduction of ROS in sleep disorders and protect hippocampal neurons from oxidative damage [9]. Notably, the fermentation process further releases bound polyphenols from *Moringa* leaves and generates novel active peptides through microbial metabolism, which provides a new idea for the development of highly effective and low-toxicity sleep modulators [10].

Recent studies have found a bidirectional regulatory relationship between the sleep–wake cycle and brain metabolic dynamics and gut flora composition: cerebrospinal fluid β-amyloid clearance is elevated by 60% during slow-wave sleep, and gut flora metabolites can affect hypothalamic orexin neuronal activity via the vagal pathway [11]. Preclinical evidence suggests that the imbalance of the anaplasmosis/thick-walled bacillus phylum ratio in sleep-deprived mice exacerbates blood–brain barrier permeability, contributing to lipopolysaccharide (LPS) entry into the brain to induce neuroinflammation [12]. Based on this, the present study was the first to systematically investigate the sleep-promoting effects of fermented extract of *Moringa oleifera* leaves (FM) on C57 mice and to reveal its multi-targeted mechanism of action through metabolomics, network pharmacology, molecular docking, and 16S rRNA sequencing techniques. This study not only provides a theoretical basis for the improvement of sleep disorders by natural products, but also lays the foundation for the development of precise intervention strategies based on the microbe-gut–brain axis (MGB axis).

## 2. Materials and Methods

### 2.1. Experimental Materials

*Moringa oleifera* leaves: Yunnan Tianyou Science and Technology Development Company (Kunming, China), the leaves were sourced from *Moringa oleifera Lam.* (voucher specimen No. MO-YN2022-01) authenticated by the Kunming Institute of Botany, Chinese Academy of Sciences. Morphological (leaf shape, flower characteristics) and genetic (ITS sequencing) identification confirmed the species; Sodium barbital, sodium pentobarbital: Kunming Mona Biotechnology Company (Kunming, China); GDMCC 1.1798 *Lactobacillus rhamnosus*, GDMCC 1.730 *Lactobacillus yoelii*, GDMCC 1.412 *Lactobacillus acidophilus*, DMCC 1.1796 *Lactobacillus fermentum*, GDMCC 1.410 *Lactobacillus casei*, GDMCC 1.2685 *Lactobacillus plantarum*, GDMCC 1.1805 *Lactobacillus rohita*, GDMCC 1.206 *Bifidobacterium shortum*, GDMCC 1.986 *Lactobacillus salivarius*, GDMCC 1.520 *Bifidobacterium longum*, GDMCC 1.791 *Lactobacillus swissii*, GDMCC 1.169 *Bifidobacterium animalis*: Guangdong Provincial Microbial Culture Preservation Center (Guangzhou, China); γ-aminobutyric acid standard: Huaxi Biotechnology Co., Ltd. (Shanghai, China); H_3_BO_3_, NaClO: Kunming Mona Biotechnology Co., Ltd. (Kungming, China); C_6_H_5_OH: Shanghai Yuanye Biotechnology Co., Ltd. (Shanghai, China); MRS medium, Bifidobacterium agar medium, Brain and Heart Leachate agar medium, Columbia agar medium: Guangdong Huankai Microbial Technology Co., Ltd. (Guangzhou, China); sodium hydroxide: Tianjin Ltd. (Tianjin, China); DZP: Wuhan Tianzhengyuan Biotechnology Co. (Wuhan, China).

### 2.2. Screening and Compounding of Bacterial Strains

Referring to the method of Molina et al. [13] the strains were inoculated in *Moringa* leaf fermentation medium, placed in a shaker for shake flask culture, and fermented at 40 ± 2 °C for 48 h. After the fermentation was completed, the fermentation broth was centrifuged (10,000 r/min, 15 min) to obtain the supernatant, and the GABA content in the supernatant was determined to screen out the strains with high GABA production. The two high GABA producing strains screened were compounded, and the effects of the compounded strains on the fermentation of *Moringa oleifera* leaves were examined by the GABA content as an index to determine the optimal compounded strains.

### 2.3. Preparation of Moringa Fermentation Extracts

Based on the results of previous studies, *Moringa oleifera* leaf powder was mixed with water at a mass ratio of 1:15 in a fermenter, sterilized at a high temperature (100 °C, 15 min) and cooled. Subsequently, the fermenter was inoculated with 0.03% of the total fermentation broth mass and fermented at 40 ± 2 °C for 48 h. After completion of the fermentation process, the mixture was filtered and the supernatant was collected for vacuum freeze drying to obtain the fermented Moringa oleifera leaf extract powder.

### 2.4. Targeted Quantification of Amino Acids

In order to investigate the changes in amino acid content before and after the fermentation of *Moringa* leaves, the present study was carried out with reference to the method of Zhang et al. [14] for the targeted quantification of amino acids by liquid chromatography. The chromatographic conditions utilized an Acquisition UPLC^®^ BEH C18 column (2.1 × 100 mm, 1.7 μm, Waterside (Warrington, UK) ) with an injection volume of 5 μL, a column temperature of 35 °C, a mobile phase consisting of 50% methanol water (0.1% formic acid) and a mobile phase B consisting of 10% methanol water (0.1% formic acid). Mass spectrometry conditions included an electrospray ionization (ESI) source in positive ion mode. The ion source temperature was set to 500 °C, the ion source voltage was set to 5500 V, the collision gas was set to 4 psi, the curtain gas was set to 40 psi, and both the nebulizer and auxiliary gases were kept at 50 psi. Scanning was performed using multiple reaction monitoring.

### 2.5. Animals and Experimental Design

Forty 6-week-old SPF-grade male ICR mice, weighing 18–22 g, were used in this study (Beijing Specific Bio-Technology Co. (Beijing, China)) They were divided into four groups (*n* = 10) using the completely randomized grouping method: a blank control group (CON, 0.9% saline), a low-dose fermented *Moringa oleifera* leaves group (FM-L, 50 mg/kg-BW), a high-dose fermented *Moringa oleifera* leaves group (FM-H, 200 mg/kg-BW), and a positive control diazepam group (DZP, 2 mg/kg-BW). All mice were housed in an SPF-grade barrier environment (temperature 24 ± 1 °C, humidity 50 ± 10%, 12 h/12 h light/dark cycle), with ad libitum intake of irradiated sterilized chow (AIN-93G formulation) and sterile water, and bedding was changed every 48 h. Acclimatization feeding was carried out for 7 days before the experiment, at the end of which 0.1 mL/20 g-BW was administered by gavage at a fixed time of day (09:00–10:00) for 28 consecutive days of intervention. The study strictly followed the approval criteria of the Experimental Animal Ethics Committee of Yunnan Agricultural University (Approval No. 202209012) and implemented animal welfare protection measures according to the ARRIVE 2.0 guidelines.

### 2.6. Sleep Behavioral Assessment

Based on the results of the pre-experiment (ED50 = 45 mg/kg), the mice were fasted for 12 h (free water) before the last administration, and sodium pentobarbital was injected intraperitoneally at a dose of 45 mg/kg-BW, with an injection volume of 0.08 mL/10 g-BW. The disappearance of the turning reflex (the mice could not be reset voluntarily within 60 s after being placed in the supine position) was regarded as a marker for the onset of sleep, and the recovery of the reflex was regarded as the end point of arousal, and the total duration of sleep was recorded. Individuals failing to fall asleep within 15 min post-injection (defined as not reaching Racine sleep classification ≥ stage III) were excluded, resulting in a final effective sample size of six mice per group [15].

The subthreshold hypnotic dose of 28 mg/kg-BW was determined by pre-experimentation, and sodium pentobarbital (28 mg/kg-BW) was injected intraperitoneally 30 min after the final administration, and the number of animals that fell asleep within 30 min was recorded to calculate the sleep incidence (%). This experiment was used to assess the synergistic effect of the drug on central inhibitors.

Sodium barbiturate (260 mg/kg-BW, 0.08 mL/10 g-BW) was injected after the final administration, and the times from the administration of the drug to the disappearance of the flip-flop reflex was recorded, which reflects the ability of the drug to modulate the rate of sleep initiation. All behavioral experiments were conducted in a double-blind method, with data recorded synchronously by two independent observers, and the differences between groups were verified by Cohen’s Kappa coefficient (κ > 0.85). Hippocampal tissues were rapidly isolated (operated on ice and snap-frozen with liquid nitrogen) and cecum contents were aseptically collected (stored at −80 °C) within 30 min after the final drug administration for subsequent metabolomics and gut microbiology analyses.

### 2.7. Measurement of Neurotransmitters

Measurement was performed according to the instructions of ELISA kits for 5-HT, GABA and Glu: the kits were equilibrated at room temperature for 20 min; the wells were set up according to the number of standards and samples, and the concentration of standard S0 was 0, which could be used as the zero point of the standard curve, and the concentration of S5 was the highest concentration, which could be used as the highest point of the standard curve; 50 μL of standard solution was added into the standard wells accurately. We diluted the sample to be tested five times with sample diluent and added 50 μL into each well; except for the S0 well, we added 100 μL of horseradish peroxidase-labeled antibody working solution into each reaction well, sealed the plate, and incubated at 37 °C for 60 min; End of incubation. We shook off the liquid in the wells of the plate and patted it dry on a piece of absorbent paper; we added 350 μL of washing solution/well, let it stand for 1 min, and then repeated the plate washing five times; we added 50 μL each of substrate A and B to each well, and incubated for 15 min at 37 °C to avoid light. At the end of incubation, we added 50 μL of termination solution to each well, measured the OD value of each well at 450 nm within 15 min, and saved the data.

### 2.8. Brain Tissue Metabolomics Analysis

Referring to the method of Presset et al. [16], hippocampal tissue samples (about 30 mg) were ground with liquid nitrogen and added into the pre-cooled extraction solution (methanol:acetonitrile = 1:1, containing 0.1% formic acid); the cells were broken by tissue homogenizer (−10 °C, 50 Hz, 6 min), extracted by low-temperature ultrasonication (5 °C, 40 kHz, 30 min), and centrifuged (13,000× *g*, 30 min) after 30 min of resting at −20 °C. The metabolite analysis was performed on an ultra-performance liquid chromatography-time-of-flight mass spectrometry system (UPLC-TripleTOF 5600+, AB SCIEX (Framingham, MA, USA)) with a Waters BEH C18 column (Waters Company, Milford, MA, USA) (100 mm × 2.1 mm, 1.7 μm), and the mobile phases of A (95% water + 5% acetonitrile + 0.1% formic acid) and B (47.5% acetonitrile + 47.5% isopropanol + 5% water + 0.1% formic acid) were used, with a column temperature of 40 °C and an injection volume of 3 μL. Mass spectrometry parameters: ion source voltage 5.5 kV, nebulizing gas temperature 550 °C, collision energy 35 eV. The raw data were converted to mzXML format by ProteoWizard, and the peaks were extracted, aligned and normalized with the HMRS algorithm by XCMS normalized, and matched with HMDB and METLIN databases. Multivariate statistical analysis and metabolic pathway enrichment analysis were performed by the Majorbio Cloud Platform (v2.0), with significance thresholds set at VIP > 1.0 and *p* < 0.05. All experimental steps were set up with quality control samples (QC, mixing equal amounts of each sample supernatant) to monitor system stability.

### 2.9. Web-Based Pharmacology

The metabolomics data-identified components of FM were initially screened using the Traditional Chinese Medicine Systems Pharmacology Database and Analysis Platform (TCMSP). At the same time, the SWISS target prediction platform was used to find and screen the predicted active ingredient targets, and Uniprot was used to correspond to the standard names of the screened proteins. Using “Insomnia” as the keyword, we searched Gene Cards, OMIM, TTD and DrugBank databases for disease targets, and then plotted the data into a Wayne diagram to obtain all the targets of insomnia.

The intersection of the FM active ingredient target and the sleep aid target was taken as the possible target of FM sleep aid, and the relationship between the intersection of the ingredient and disease target and the screened active ingredient was imported into Cytoscape 3.6.1 software to make the ingredient–target relationship network diagram. We used the intersected targets for PPI interaction analysis, and obtained the PPI protein interaction network map, then imported the file into Cytoscape’s Bisogenet for PPI protein interaction network construction. CytoNCA was used to screen the protein network for core targets and obtain the key targets of FM sleep support.

Gene Ontology (GO) mainly defines and describes the gene and protein functions of each species in terms of Biology Process (BP), Cellular Component (CC) and Molecular Function (MF). Kyoto Encyclopedia of Genes and Genomes (KEGG) pathway enrichment analysis is commonly used to elucidate the role of target proteins in signaling pathways. In this study, GO enrichment analysis and KEGG pathway analysis of shared targets were performed using the Bioinformatics database, and the corresponding targets in the FM were directly mapped to the pathways and visualized in a bubble map.

### 2.10. Molecular Docking

The core of molecular docking is to predict and study the interactions between molecules, especially the cooperation and interactions between small molecules (e.g., drug molecules) and large molecules (e.g., proteins) [17]. In this study, combining molecular docking and the aforementioned bioinformatics analysis can help us comprehensively assess the therapeutic potential of the active ingredients in the FM aid and provide a scientific basis for its further research and clinical application. The 2D structures of the active ingredients were downloaded using the PubChem database (https://pubchem.ncbi.nlm.nih.gov/), converted to 3D structures using Chem3D software (Version 14.0.0.17), and saved in mol2 format with energy minimization operations. The proteins were extracted from the RCSB PDB database (https://www1.rcsb.org/); the source was set as “homo spacies”, and the 3D structures of the target proteins were obtained and saved in PDB format. The proteins were dehydrated and residues were removed using PyMOL software (Version 3.0); the molecular energies were minimized using spdbv4.1.0 software, and hydrogen atoms were added to the proteins and ligands.

### 2.11. 16S rRNA Gene Sequencing Analysis

An appropriate amount of appendix content sample was melted at 4 °C and mixed homogeneously with pre-cooled methanol/acetonitrile/water solution (2:2:1, *v*/*v*). The samples were subjected to low-temperature sonication (30 min, −20 °C) and centrifuged at 14,000× *g* for 10 min at 4 °C for 20 min, and then the samples were transferred to a vacuum desiccator for drying. The samples were reconstituted with aqueous acetonitrile for mass spectrometry analysis, and the supernatant was centrifuged for subsequent experiments. A fecal DNA isolation kit (MoBio Laboratories, Carlsbad, CA, USA) was used to isolate genomic DNA; the 16S rRNA V3-V4 region was amplified by an ABIGeneAmp^®^9700 PCR instrument (Perkin Elmer Company, Waltham, MA, USA), and the PCR products were analyzed by Quantifluorescence™™-ST Blue Fluorescent Quantification System (Beijing Qunxiaokeyuan Biotechnology Co., Ltd., Beijing, China). PCR products were quantified by Quantifluorescence™-ST Blue Fluorescent Quantification System (Promega, Madison, WI, USA) for quantification. Primers were designed as 338F and 806R. Sequencing libraries were generated using a TruSeq^®^ DNA PCR-free sample preparation kit (Illumina, San Diego, CA, USA). The above bioinformatics analysis was provided by the Majorbio cloud platform (Shanghai Meiji Biomedical Technology Co., Ltd., Shanghai, China).

### 2.12. Data Analysis

The study was statistically analyzed using SPSS software (version 26.0). Analytical validity was confirmed through LC-MS/MS with linear calibration curves (0.1–100 µg/mL, R^2^ > 0.99) and excellent reproducibility (intra-/inter-day CVs < 15%). Group differences were assessed by one-way ANOVA followed by Tukey’s post hoc test, with inter-rater reliability for behavioral data confirmed by Cohen’s κ > 0.85. Results were considered statistically significant at *p* < 0.05 (* < 0.05, ** < 0.01, *** < 0.001).

## 3. Results

### 3.1. Screening and Compounding of Bacterial Strains

**Figure 1:**
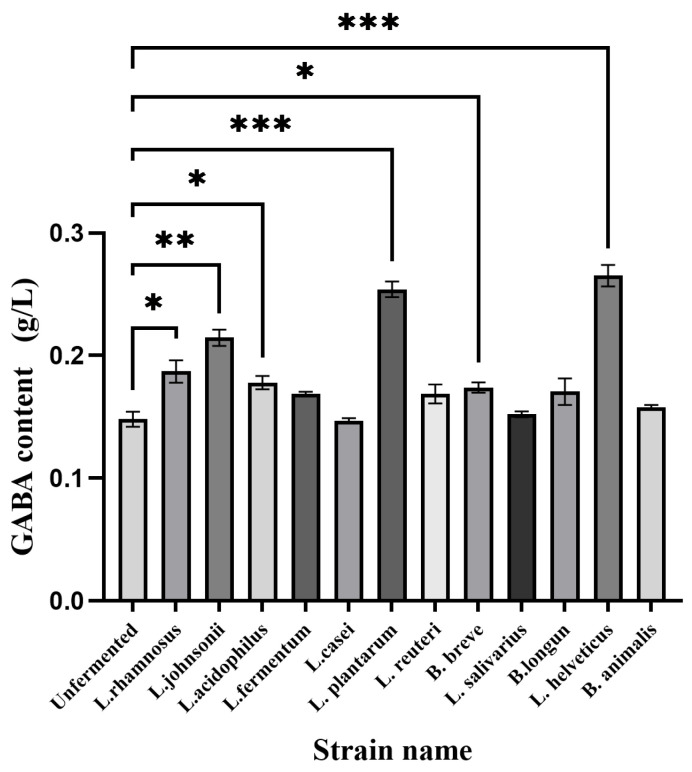
Screening results of GABA-producing bacteria (* *p* < 0.05, ** *p* < 0.01, *** *p* < 0.001).

The GABA standard curve was established with GABA concentration as the horizontal coordinate and absorbance (OD value) as the vertical coordinate, as shown in Figure S1A. The regression equation of the GABA standard curve was y = 2.31857x − 0.00413, and the regression coefficient R^2^ = 0.99906, which indicated that there was a good linear relationship between GABA concentration and absorbance. The GABA content was determined in the unfermented group and 12 strains of the *Lactobacillus* fermentation group. The difference between the GABA content in different groups and the unfermented group was analyzed by one-way ANOVA, and the results are shown in Figure 1. Compared with the unfermented group, among the 12 strains of *Lactobacillus*, *Lactobacillus rhamnosus*, *Lactobacillus acidophilus*, and *Bifidobacterium shortum* fermentation significantly increased the GABA content (*p* < 0.05); *Lactobacillus cohnii* very significantly increased GABA content (*p* < 0.01), *Lactobacillus plantarum* and *Lactobacillus swissii* extremely significantly increased GABA content (*p* < 0.001), whereas the other strains were not significantly different (*p* > 0.05). Among them, *Lactobacillus plantarum* and *Lactobacillus swissii* fermented GABA concentrations up to 0.254 g/L and 0.265 g/L, so these two strains were used as fermentation strains for subsequent experiments.

As shown in Figure S1B–D, the ratio of composite strains, inoculum amount, fermentation temperature, feed-to-liquid ratio and fermentation time have important effects on the content and quality of the products in the fermentation process. Under the optimal fermentation conditions, the GABA content reached 0.272 g/L, which was 1.67 times higher compared with the unfermented content of 0.163 g/L.

### 3.2. Quantitative Results of Amino Acids

The results of quantitative amino acid analysis of moringa samples before and after fermentation are shown in Table 1, indicating that 20 amino acids were identified in unfermented *Moringa oleifera* leaves with a total content of 43,112.62 ± 591.54 µg/g, of which nine essential amino acids accounted for 33.97% (14,644.47 ± 302.86 µg/g), with aspartic acid, alanine, and phenylalanine predominant. The total amino acid content of the fermented extract of *Moringa oleifera* leaves (FM) increased significantly to 46,058.20 ± 845.53 µg/g (*p* < 0.05), while the percentage of essential amino acids decreased to 31.07% (14,311.82 ± 212.05 µg/g). Notably, asparagine (23.13%), glutamine (9.16%), alanine (7.80%), phenylalanine (7.39%) and arginine (7.20%) became the major components of FM, which together accounted for 54.68% of the total amino acids. This change suggests that microbial activity may preferentially consume some of the essential amino acids while enriching non-essential amino acids.

The amino acid profiles of *Moringa* leaves before and after fermentation were subjected to Principal Component Analysis (PCA) and Orthogonal Partial Least Squares Discriminant Analysis (OPLS-DA), respectively, and the results are shown in Figure S1A,B. The PCA score plots showed that PC1 (84.5%) + PC2 (10%) explained a total of 94.5% of the variance, which suggests that the first two principal components are sufficiently reflective of the main characteristics of the data. The OPLS-DA results show that the T score explains 83.9%, indicating that the model has a strong ability to capture between-group differences. Among them, R^2^ was 0.945 (*p* < 0.05) and Q^2^ was 0.998 in the model validation, indicating that the established model had good fit and robustness and effectively elucidated the differences in amino acid content before and after fermentation. The changes in amino acid content between the two groups were visualized by a color gradient-based clustering heat map. As shown in Figure S1C, the fermentation of *Moringa* leaves resulted in an increase in the content of Tyr and Phe, which are associated with the synthesis of the neurotransmitter dopamine and affect the sleep–wake cycle. Contrasting metabolites, such as Arg, Orn, Gln, and Glu, may be the focus of subsequent mechanistic studies.

### 3.3. Improvement of Sleep Quality by FM in Mice

**Figure 2:**
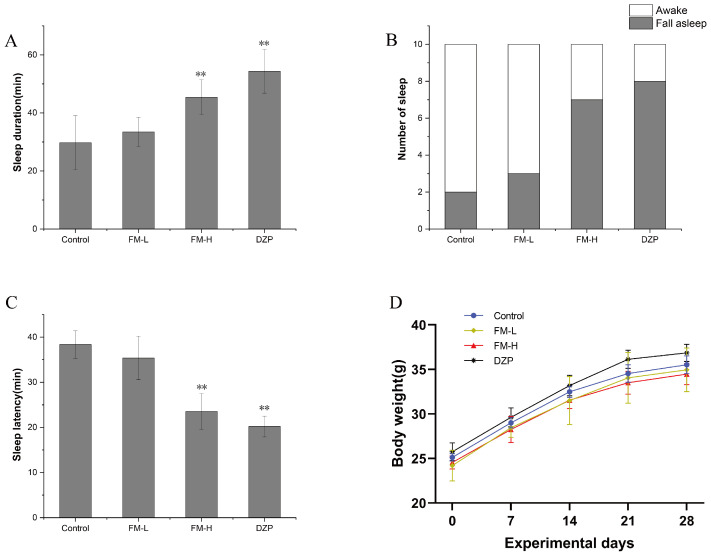
Pentobarbital sodium induced sleep experiment. (**A**) The sleeping time. (**B**) Incidence of sleep in mice. (**C**) Sleep latency of mice. (**D**) Weight growth trend (** means *p* < 0.01).

After 4 consecutive weeks of gavage treatment, none of the mice in the experimental groups showed direct sleep induction during the 60-min observation period after the last administration, indicating that the fermented extract of FM itself does not have an acute sedative effect. In the sodium pentobarbital synergistic experiment (Figure 2A), it was found that the sleep duration of experimental animals in the FM-L and FM-H group was (33.44 ± 5.04) min and (45.42 ± 5.95) min, respectively, which was 12.48% and 52.77% longer than that of the control group (29.73 ± 9.29) min, respectively. Notably, the FM-H group demonstrated a significant dose-dependent potentiation (*p* < 0.01) compared to the control ratio, while the prolongation effect in the FM-L group did not reach statistical significance (*p* > 0.05).

In the subthreshold dose of the sodium pentobarbital model (Figure 2B), both the FM-H group and the positive control diazepam group (DZP) significantly elevated sleep onset (*p* < 0.01), whereas the intervention effect of the FM-L group was not statistically different from the control group. This dose–response relationship suggests that FM may exert a sleep-aiding effect by enhancing the function of the central inhibitory neurotransmitter system. Sleep latency analysis showed (Figure 2C) that the sleep initiation time was significantly shorter (*p* < 0.01) in the FM-H group (23.53 ± 3.93 min) and the DZP group (20.19 ± 2.31 min) compared to the control group (38.37 ± 3.03 min), whereas the trend of shortening in the FM-L group (35.38 ± 33.82 min) did not reach statistical significance. Notably, there was a large standard deviation in the data of the FM-L group, suggesting that individual response heterogeneity may have influenced the statistical efficacy of the low-dose group.

During the 4-week intervention, the experimental animals in each group were in good growth condition, and no abnormalities in feeding and daily activities were observed (Figure 2D). Although the final body weight of the FM-H group was slightly lower than that of the control group, and that of the DZP group was slightly higher than that of the control group, the comparison between the groups showed that none of the body weight changes were statistically different. This result indicated that FM did not significantly affect the normal growth and development of the experimental animals within the dose range designed for this study.

### 3.4. Effect of FM on Hypothalamic Neurotransmitters in Mice

Neurotransmitters 5-HT, Glu and GABA were detected in mice in the normal control group, FM-L group, FM-H group, and DZP group, respectively, and the data of each group were expressed as mean ± standard deviation. Table 2 shows the levels of neurotransmitters in the hypothalamus of mice compared with the control group; 5-HT levels were significantly increased in both the FM-H group (*p* < 0.05) and the DZP group (*p* < 0.01), with the greatest increase in the DZP group. The level of Glu decreased in a dose-dependent manner, and FM-H group and DZP group were significantly lower than the control group (*p* < 0.01). GABA content was significantly increased in all treatment groups, with FM-H, and DZP groups increasing by 26–40% compared with the control group, respectively, with the DZP and FM-H groups showing the strongest pro-GABA release effects (*p* < 0.01). It is worth noting that the regulatory effects of the FM-H group on the three indexes were close to or reached the level of the GABA-positive control group, suggesting that FM-H may exert biological effects through the synergistic regulation of monoamine and amino acid neurotransmitter systems. Glu is the most important excitatory neurotransmitter in the central nervous system, while GABA is an inhibitory neurotransmitter, and the balanced relationship between Glu and GABA is the basis for ensuring the maintenance of the normal physiological functions of the brain [18]. Both FM-H and DZP can help sleep by enhancing inhibitory regulation, and the effect of DZP is more powerful. The Glu/GABA ratio in the FM-L and FM-H groups decreased from 3.608 to 2.527, with a decrease of 30%, which indicated that the elevated dose of FM significantly strengthened inhibitory regulation. The ratio of the FM-H group was 40.2% lower than that of Control group, and that of the DZP group was 50.3% lower than that of the Control group. The ratio of the DZP group was significantly lower than that of the FM-H group, indicating that DZP regulated the excitation–inhibition balance more thoroughly, which was consistent with its classical mechanism of directly enhancing the GABA receptor. On the other hand, the FM-H group, on the basis of maintaining significant efficacy, demonstrated the therapeutic advantage of “precise balance” through the synergistic regulation of multiple transmitters, smoother neuroinhibition (low volatility of Glu), and potentially better safety (avoiding over-activation of GABA).

### 3.5. Detection and Analysis of LC-MC

The mechanism of FM regulation on the mouse central nervous system was systematically revealed by untargeted metabolomics. As shown in Figure S1, PCA showed that all sample points were located within the 95% confidence ellipse, which verified the normal distribution property of the data. Notably, the FM-L group and the control group showed partial overlap on the PC1 axis (R^2^X = 0.42), while both the FM-H group and the positive control DZP group exhibited significant spatial separation (R^2^X = 0.58), suggesting that the high-dose intervention induced more significant metabolic reprogramming. The dose effect was further confirmed by PLS-DA, in which the Q^2^ value of the FM-H group amounted to 0.86, which was significantly higher than that of the FM-L group (Q^2^ = 0.51), suggesting that the high-dose treatment could distinguish metabolic profiles between groups more effectively.

**Figure 3:**
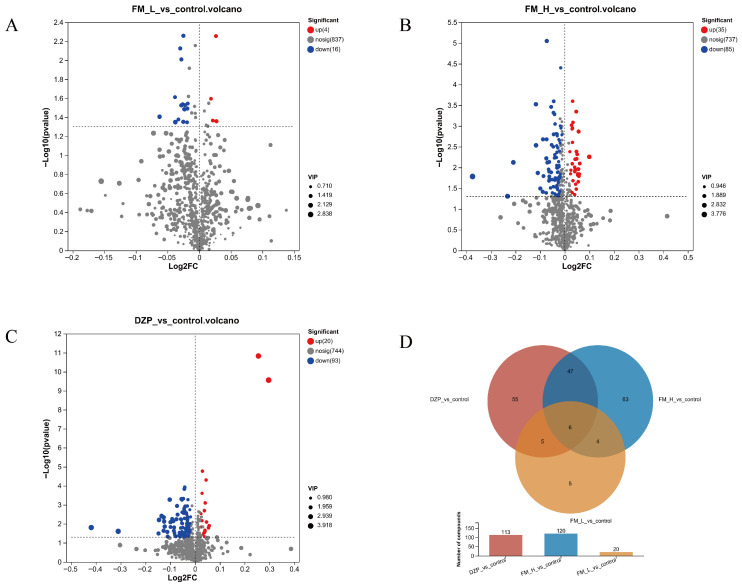
(**A**–**C**) Volcano plots of different metabolites (positive and negative ion binding) among different groups. (**D**) Venn diagram of differential metabolites.

**Figure 4:**
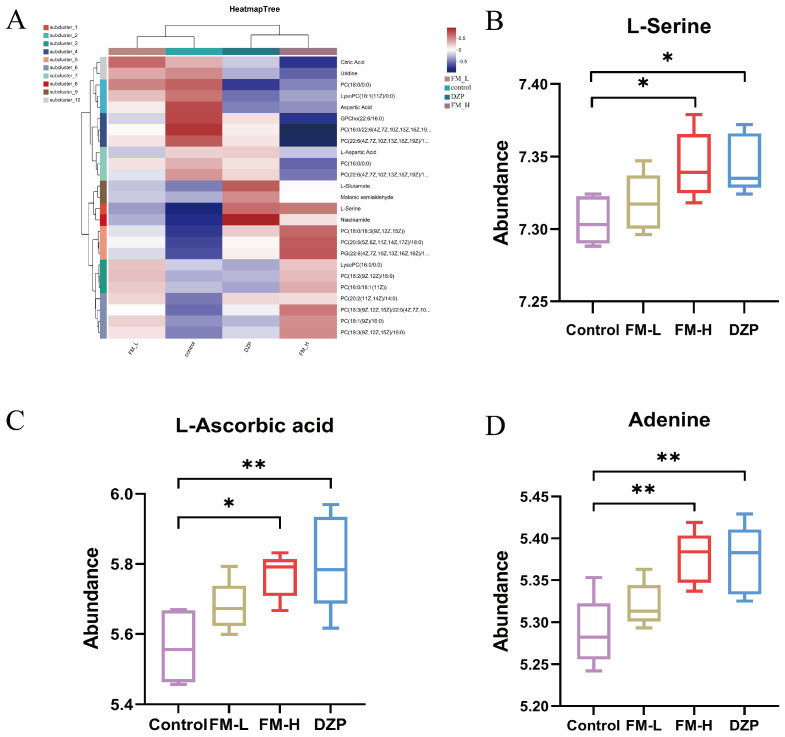
(**A**–**C**) Heat maps of the top 25 differentially abundant metabolites in different groups. (**D**) Differentially abundant metabolites (* *p* < 0.05, ** *p* < 0.01).

Based on a multi-criteria screening strategy (*p* < 0.05, VIP > 1, |log2FC| > 0.58), sleep-related metabolites were rigorously screened. Volcano plot analysis revealed that the FM intervention triggered significant metabolic perturbations (Figure 3A–C). Compared with the control group, 120 differential metabolites were identified in the FM-H group, of which 35 metabolites were up-regulated and 85 were down-regulated, which was about three times the number of the FM-L group and shared 47 core metabolites with the DZP group (Figure 3D). To deeply elucidate the intrinsic association between different samples and the differences in metabolic expression, we performed hierarchical cluster analysis (HCA) on the expression levels of the top 25 significantly different metabolites (Figure 4A), which showed that the FM-H group specifically up-regulated L-serine and L-glutamate, down-regulated neurotransmitter precursors such as L-aspartate, and also up-regulated PC (18:0/18:3 (9Z, 12Z, 15Z), and down-regulated membrane phospholipid components such as GPCho, PC (18:0/0:0) (Figure 4B–D). This series of results strongly suggests that FM may have an impact on sleep regulation through the precise regulation of neurotransmitter synthesis and membrane stability. At the molecular level, changes in neurotransmitter precursors directly alter the amount of neurotransmitter synthesis and affect inter-neuronal signaling [19], whereas changes in membrane phospholipid composition affect the fluidity and stability of cell membranes, influence the functional state of neuronal cells, and ultimately synergistically exert an effect on the sleep regulatory pathway [20].

**Figure 5:**
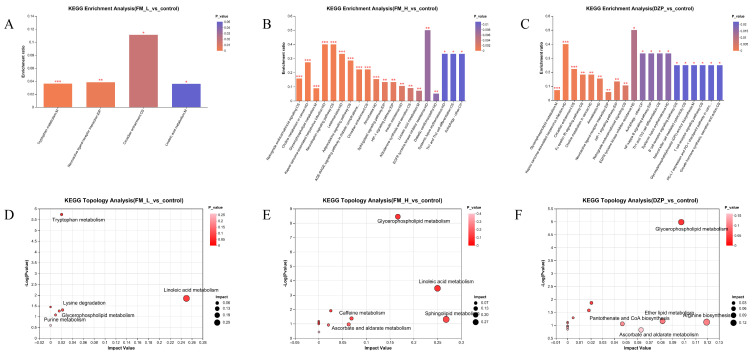
KEGG pathway analysis. (**A**–**C**) Analysis of metabolite enrichment pathways in different groups: (**A**) FM-L vs. Control; (**B**) FM-H vs. Control; (**C**) DZP vs. Control. (**D**–**F**)Analysis of KEGG pathway topology in different groups: (**D**) FM-L vs. Control; (**E**) FM-H vs. Control; (**F**) DZP vs. Control (* *p* < 0.05, ** *p* < 0.01, *** *p* < 0.001).

The dose-dependent pathway regulation was clearly characterized by KEGG enrichment analysis (Figure 5A–F). In the FM-L group, tryptophan metabolism as well as neuroactive ligand–receptor interaction pathways were mainly affected. In terms of molecular mechanisms, the alteration of tryptophan metabolism, as a precursor of 5-HT, is likely to be closely related to the regulation of the 5-HTergic system, and the 5-HTergic neurons, which are widely distributed in the CNS, play a key role in the physiological processes such as the sleep–wake cycle and emotion regulation, etc. The effect of FM-L on tryptophan metabolism may be through the alteration of 5-HT synthesis and release, which in turn may affect the 5-HT-agonist pathway (Figure 5A–F). For the FM-H group, the retrograde endogenous cannabinoid signaling and sphingolipid metabolic pathways were significantly perturbed. In the retrograde endogenous cannabinoid signaling pathway, the cannabinoid receptor, CB1R, is widely expressed in the presynaptic membrane of neurons in the CNS, and the modulation of this pathway by the FM-H group suggests that it may affect the function of neural circuits through activation of the CB1R, regulation of neuronal synaptic transmission, and alteration of neurotransmitter release. Meanwhile, perturbation of the sphingolipid metabolic pathway is closely linked to neuroprotective mechanisms. Sphingolipids and their metabolites are important in maintaining the structural and functional integrity of neuronal cell membranes [21], and the effect of the FM-H group on this pathway may be indirectly involved in the process of sleep regulation by enhancing neuroprotection and improving the survival environment of neurons. The DZP group specifically regulates the HIF-1 signaling pathway, which is related to its known function of hypoxic stress regulation. HIF-1, as a transcription factor, is activated under hypoxic conditions and regulates the expression of a series of genes, which are involved in cellular adaptive responses [22]. DZP’s regulation of the HIF-1 signaling pathway may affect neuronal metabolism and function through regulating the cellular response to hypoxic stress and thus play an important role in sleep regulation and function. Topological analysis showed that the FM-H group had the most significant regulatory effects on arachidonic acid metabolism (Impact = 0.38) and purine metabolism (Impact = 0.31). Arachidonic acid metabolism produces a variety of bioactive substances, such as prostaglandins and leukotrienes, which are involved in inflammatory responses, neuromodulation, and other processes, and are closely related to sleep–wake cycle regulation [23]. Purine metabolites, such as adenosine, are important endogenous sleep-promoting substances. Changes in their metabolic processes can directly affect sleep homeostasis [24]. The regulation of these two pathways by the FM-H group suggests that it may accurately regulate the molecular network of the sleep–wake cycle through the modulation of arachidonic acid metabolite as well as purine metabolite production and metabolism, and realize the effective regulation of sleep.

### 3.6. Network Pharmacology Predicts Sleep-Aiding Targets

The hypothalamic tissues of the blank and experimental groups were analyzed by LC-MS/MS and screened for key targets of brain entry components according to the method in Section 2.9. The results showed that there were 10 FM brain-entry components (Table 3), which were 3,6,7-Trihydroxy-4′-methoxyflavone 7-rhamnoside, Cinncassiol A, Palmitoyl Ethanolamide, Nepetaside, 8(R)-Hydroperoxylinoleic acid, Dodecyl Hydrogen Sulfate, Melleolide, Melatonin, (S)C(S)S-S-Methylcysteine sulfoxide, and Uric Acid.

**Figure 6:**
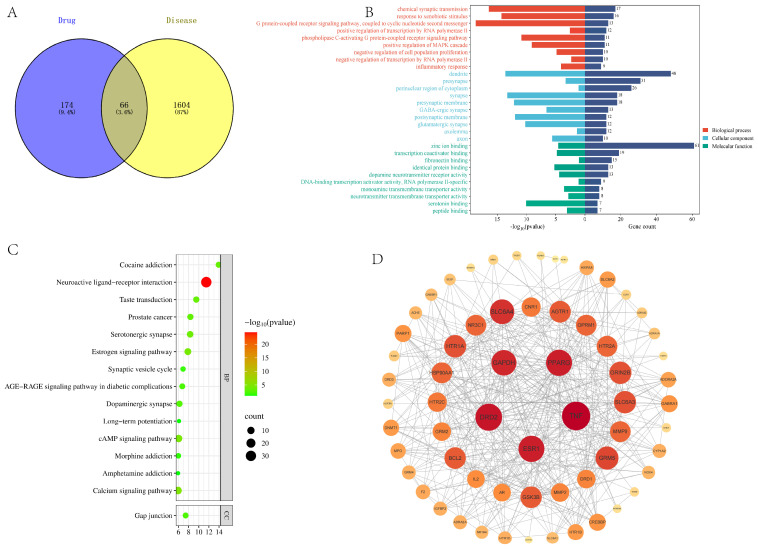
(**A**) Wayne diagram. (**B**) GO analysis. (**C**) Bubble diagram. (**D**) PPI molecular interaction.

The potential targets of these active substances were predicted and screened using a drug database, and after integration and elimination of duplicates, 1604 potential targets of action related to the potency of FM were identified. The disease database was used to collect and search for insomnia diseases, and 174 biological targets associated with the disease were obtained by integrating the target data screened in the database and eliminating the duplicated genes and leaving only one. The 66 drug–disease common targets were obtained from the intersection of active ingredients and disease targets, and the Venny 2.1.0 online mapping tool was utilized to draw a Wayne diagram, as shown in Figure 6A.

The PPI network of drug–disease common targets was analyzed and the disconnected nodes were hidden, involving 66 nodes and 405 edges. The intersected targets were analyzed for biological enrichment using the DAVID toolbox, and were ranked according to the *p*-value of the GO functional classification enrichment items, where a lower *p*-value implies a higher level of enrichment. Bar charts were created for the top 10 items in terms of BP, CC and MF, as shown in Figure 6B. The top 15 KEGG pathways with high enrichment levels were also selected based on *p* values. The enrichment analysis showed that the active ingredients in FM were mainly concentrated in important biological pathways such as Neuroactive ligand–receptor interaction, the Estrogen signaling pathway, the cAMP signaling pathway and the Calcium signaling pathway in regulating insomnia, which are closely related to physiological processes such as neuromodulation, cell survival, proliferation and lipid metabolism. This finding elucidates the complex mechanism of action of FM in the treatment of insomnia, and emphasizes its wide-ranging effects on multiple components, multiple targets and numerous biological pathways.

Using Cytoscape software, information on FM active ingredients and key disease targets targeted by the drugs, including data related to the first 15 KEGG pathways, was summarized and entered in order to construct a graphical representation of the drugs, active ingredients, targets, diseases, and pathways, as shown in Figure 6D. This figure reveals the multi-component, multi-target and multi-pathway action characteristics of the FM sleep aid. Based on the existing studies and target relevance screening, it was determined that GABRA1, HTR1A, GRM5, HTR1B, HTR2A, GABBR1, and GRM2 could be used as the key targets of the FM sleep aid.

### 3.7. Molecular Docking Validation

In order to further verify the feasibility of the FM-assisting active ingredients in assisting sleep, molecular docking was carried out using AutoDock Vina in AutoDock Tools to verify the interactions between the three active ingredients of FM and five molecules with sleep-assisting potential obtained through network pharmacology. The docking results are shown in Table 4. In molecular docking studies, the binding energy is a key indicator; in general, if the binding energy is less than 0, it indicates that the ligand molecule can spontaneously bind to the receptor protein, and if the binding energy is ≤5.0 kcal/mol^−1^, it indicates good binding. The binding conformation refers to the spatial arrangement of the ligand when it binds to the receptor, and a lower binding energy usually implies more stable binding conformations [25]. GABA exists in Hydrogen bonding with Asn414, Asp420, and Arg424 on the GBRA1 gene, and notably, it also exists with Glu277 Electrostatic with an energy of −4.3 kcal/mol^−1^; 5-HT binds to the HTR1A receptor through hydrogen bonding and hydrophobic interactions; Glu forms a hydrogen bond with Asn459 on the GRM5 gene with an energy of −4.8 kcal/mol^−1^; 5-HT exhibits Hydrogen bonding with Thr134 on the HTRIB gene with an energy of −6.3 kcal/mol^−1^.

**Figure 7:**
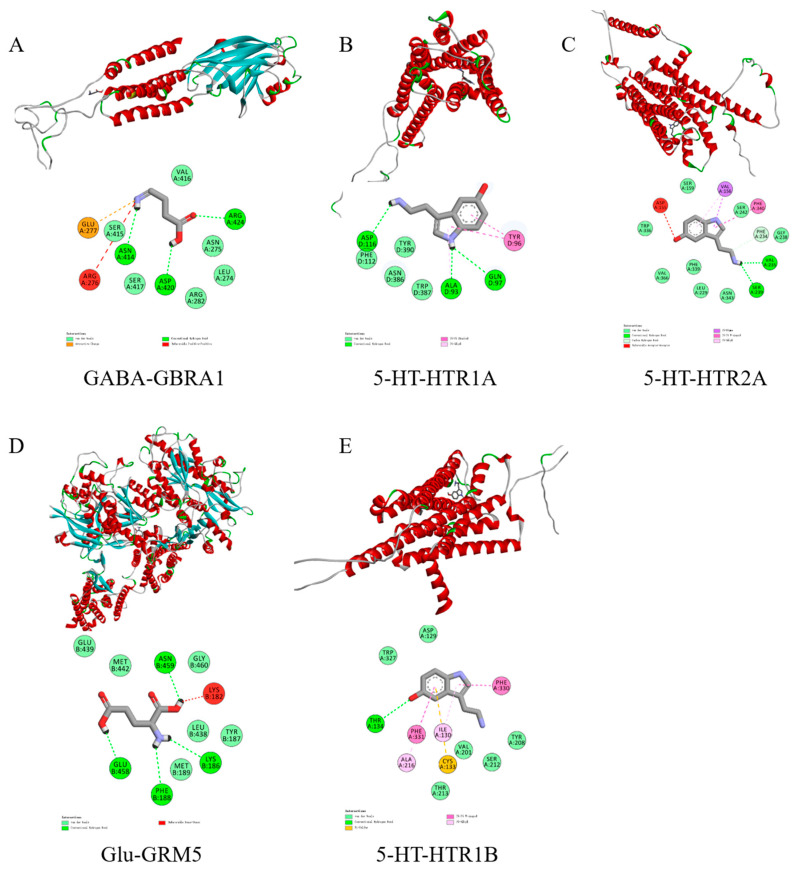
Binding of three important sleep-aiding compounds to five key target proteins by molecular docking ((**A**): GABA and GBRA1; (**B**): 5-HT and HTR1A; (**C**): 5-HT and HTR2A; (**D**): Glu and GRM5; (**E**): 5-HT and HTR1B).

The molecular docking results illustrate that the binding between the five active ingredients mentioned above and the corresponding three proteins is stable and specific, with each active ingredient forming a hydrogen bonding interaction with at least one specific site of the corresponding protein. This stable binding suggests a possible pharmacological role of these active ingredients in organisms. The visualization of molecular docking further revealed the specifics of these interactions (Figure 7). Through these detailed docking analyses, we were able to gain a deeper understanding of the mechanism of action of the active ingredients in FM, providing an important scientific basis for future research and clinical applications.

### 3.8. 16. S rRNA Analysis

**Figure 8:**
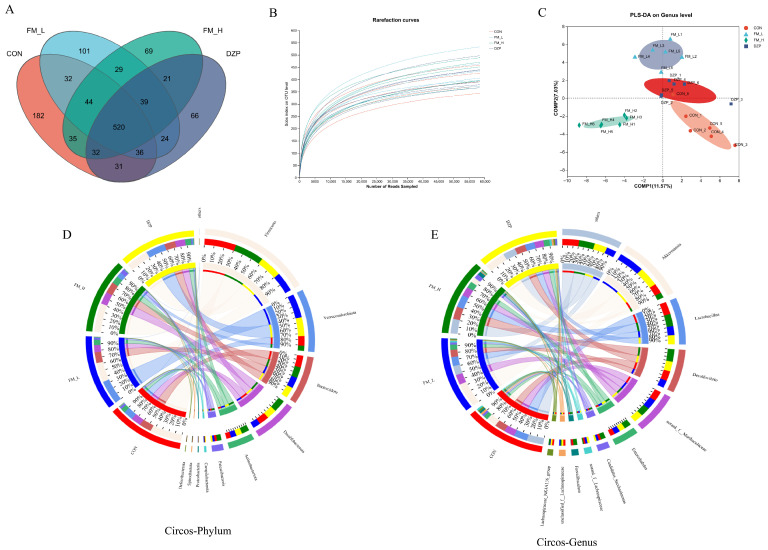
Changes in the structure of the intestinal flora in different groups of mice. (**A**) Venn diagram at the OTU level. (**B**) Species dilution curves for different samples. (**C**) PLS-DA analysis at the genus level of the intestinal flora in each group of mice. (**D**) Distribution of the four groups of microbial communities at the phylum level. (**E**) Distribution of four groups of microbial communities at the genus level.

The results of α/β diversity analysis based on 1261 Operational Taxonomic Units (OTUs) (Figure 8A–C) showed that the FM intervention was able to significantly reduce the proportion of core OTUs. The Rarefaction curve analysis further confirmed that the sequencing depth in this study was sufficient to cover the species diversity in the samples, ensuring the reliability and comprehensiveness of the data. In addition, the results of PLS-DA showed that the FM treatment group showed a significant trend of segregation on the principal component 1 axis (PC1, with 26.8% explained variance), which indicated that the structure of the intestinal flora of the FM treatment group differed from that of the other groups significantly. In contrast, the explained variance along the PC1 axis of the DZP group was 18.4%, and the FM-treated group contributed significantly more to the intergroup differences than the DZP group. This result strongly suggests that FM has a specific regulatory effect on the intestinal flora, and may exert its biological effects by changing the composition and structure of the intestinal flora.

By Circos analysis (Figure 8D–E), at the phylum level, we found that the FM-L group was able to significantly decrease the abundance of the Firmicutes, while increasing the abundance of the Verrucomicrobia. It has been shown that this phenotype of change in flora abundance is closely associated with improved metabolism of Short Chain Fatty Acids (SCFAs). As important metabolites of intestinal flora, SCFAs play an important role in maintaining the stability of the intestinal internal environment and regulating host metabolism and immune function [26]. Therefore, the altered abundance of the thick-walled and warty microflora in the FM-L group may positively affect host health by influencing the production and metabolism of SCFAs. In addition, the abundance of Proteobacteria was lower in the FM-L, FM-H, and DZP groups than in the control group, which may reflect the beneficial modulatory effect of these treatments on the homeostasis of the intestinal flora, as over-proliferation of Proteobacteria is commonly associated with intestinal inflammation and disease states.

At the genus level, the abundance of *Akkermansia* was increased 1.5-fold in the FM-H group compared to the control group. *Akkermansia* spp. is a bacterium with important physiological functions in the intestinal tract, and its increased abundance is closely associated with enhanced intestinal barrier function and suppression of neuroinflammation [27]. This result suggests that FM-H may have a protective effect on the health of the nervous system by promoting the growth and proliferation of *Ackermann*’s spp., enhancing the intestinal barrier function, and reducing the leakage of harmful substances, thereby reducing neuroinflammation. In addition, it is noteworthy that a specific enrichment of Candidatus_Saccharimonas was observed in the FM group, and this genus may be involved in the metabolic process of dietary fiber and produce metabolites with neurological activity [28]. The metabolites of dietary fiber may not only regulate the composition and function of intestinal flora, but also affect the nervous system through the gut–brain axis, which provides new clues and directions for further research on the regulatory mechanisms of FM on intestinal flora and the nervous system.

**Figure 9:**
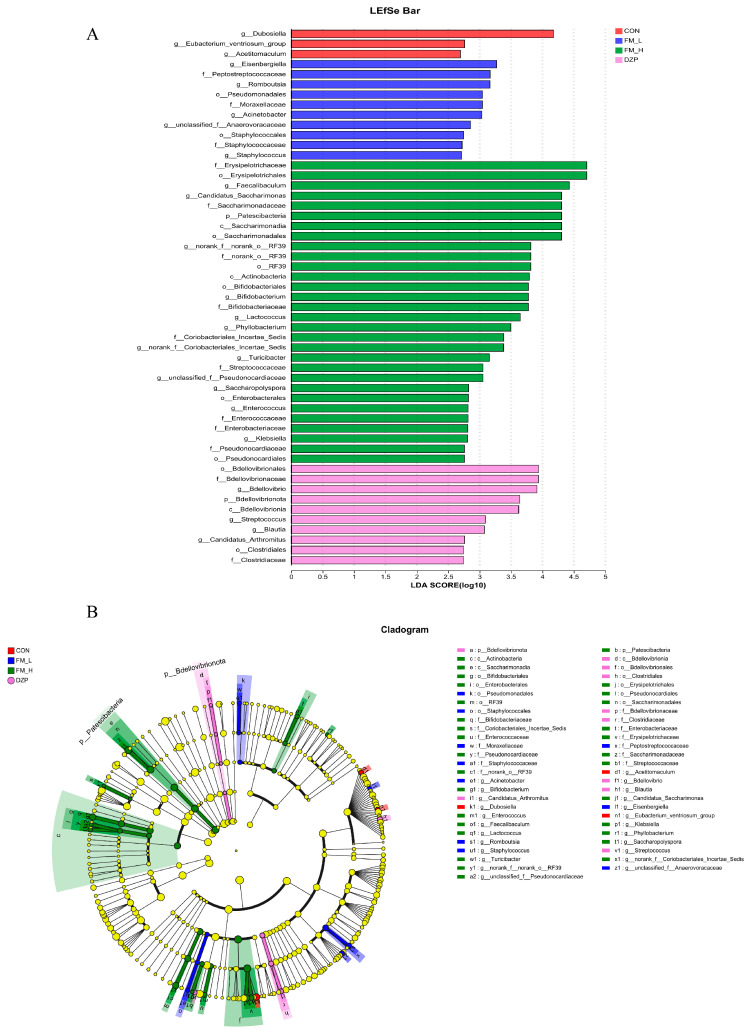
LEfSe analysis of different groups of gut flora. (**A**) Hierarchical tree diagram of LEfSe species for different groups of intestinal microorganisms. (**B**) Histogram of LDA values of different groups of intestinal microorganisms.

LEfSe results (Figure 9) demonstrated that Erysipelotrichaceae (LDA = 4.8) and Actinobacteria (LDA = 4.2) showed specific enrichment status in the FM-H group. A large number of studies have shown that these two groups of microorganisms are closely associated with the anti-inflammatory phenotype of the organism. In the regulation of inflammatory response, Danitofilamentaceae and Actinobacteria may exert their anti-inflammatory effects by regulating the activity of immune cells, the secretion of cytokines, and other pathways, thus improving the inflammatory microenvironment of the organism [29]. On the other hand, Clostridiaceae (LDA = 4.5) were significantly enriched in the DZP group. Some members of Clostridiaceae are closely related to host energy metabolism, and their over-enrichment may interfere with the normal energy homeostasis regulation mechanism of the organism, which in turn may be a potential cause of the weight gain phenomenon in the DZP group. Weight gain usually involves an imbalance between energy intake and expenditure, and the enrichment of Clostridiaceae may affect the intestinal absorption of nutrients and metabolite production, and ultimately lead to an increase in energy storage [30]. Of interest, the enrichment of Akkermansia across the dose gradient in the FM group was significantly and positively correlated with the activation of the cannabinoid signaling pathway in brain metabolomics. The cannabinoid signaling pathway is involved in the regulation of several physiological functions in the central nervous system, such as neurotransmitter release and regulation of neuronal excitability [31]. This finding implies that FM may indirectly regulate the cannabinoid signaling pathway in the brain by affecting the abundance of Akkermansia in the gut, which provides new molecular mechanism clues to understand the regulation of the nervous system function by FM.

## 4. Discussion

Sleep quality is closely linked to key physiological indicators such as cognitive function, immune response, emotional stability, and work efficiency, and has a direct and profound impact on an individual’s overall quality of life [32]. Given the systemic role that sleep plays in maintaining physical and mental health, the present study focused on exploring sleep regulators derived from natural plant sources. By analyzing the amino acid composition of fermented moringa leaves (FM) and their sleep-inducing activity, we found that FM is rich in a variety of amino acid components that are directly related to sleep regulation. For example, glycine has been demonstrated in a number of studies to significantly enhance subjective sleep quality in insomnia patients [33], while arginine can effectively balance the apoptosis phenomenon induced by sleep deprivation [34]. Of particular interest is that the fermentation process significantly increased the content of sleep-promoting amino acids such as GABA in FM, which is highly consistent with the mechanism of action of GABA in alleviating the symptoms of insomnia as verified in clinical studies.

Sleep–wake disorders are capable of triggering abnormalities in specific metabolic pathways, and this metabolic remodeling is most likely a key mechanism for their association with psychiatric disorders and various pathological states [35]. In the present study, we found that FM intervention significantly altered the metabolite profiles in the mouse brain, as evidenced by the up-regulation of the expression of key molecules such as serine, ascorbic acid and adenine. Serine, as a precursor of neurotransmitters such as glutamate and glycine, occupies a central position in the regulation of neurological disorders [36]; adenine, as an endogenous sleep homeostatic regulator, has a direct impact on the sleep cycle and quality of sleep [24]; and ascorbic acid, by virtue of its antioxidant mechanism, plays a central function in the maintenance of immune homeostasis [37], all of which suggest that FM has the potential to be a multifaceted metabolizer in mice. The above findings provide a strong metabolomic basis for the multidimensional sleep-improving effects of FM.

From the perspective of metabolic pathways, FM mainly regulates purine metabolism and tryptophan metabolism. In purine metabolism, the dynamic balance between adenosine and adenosine triphosphate (ATP) mediates the sleep-driven mechanism through purinergic receptors, and its metabolic cycle is directly related to the wake-sleep transition [38]; tryptophan metabolism becomes the core biochemical basis of sleep cycle regulation through the generation of neurotransmitters such as 5-hydroxytryptamine and melatonin. In addition, cytokines derived from arachidonic acid metabolism have been found to be involved in both immune regulation and maintenance of sleep homeostasis, and the molecular mechanism by which caffeine affects sleep by antagonizing adenosine receptors further confirms the centrality of the purine metabolism pathway in sleep regulation.

Intestinal flora analysis showed that FM intervention could significantly reduce the ratio of Firmicutes to Bacteroidetes (F/B ratio), and it has been demonstrated that an elevated ratio is positively correlated with the prevalence of anxiety and depression, as well as reduced sleep quality. Notably, the elevated abundance of Ackermannia showed a significant correlation with the FM-treated group, and the genus was able to exert gut–brain axis regulation by improving the host’s metabolic and immune responses. The data suggest that FM may indirectly affect neurotransmitter metabolism and inflammatory responses by remodeling the structure of the intestinal flora, especially the F/B ratio and probiotic abundance, thus forming a multi-targeted mechanism for sleep improvement.

## 5. Conclusions

This study demonstrates that the fermented product of *Lactobacillus plantarum* and *Lactobacillus helveticus* (1:1) exerts sleep-promoting effects in mice under controlled conditions, significantly increasing GABA (1.67-fold) and total amino acids (46,058.20 ± 845.53 μg/g) while reducing sleep latency and prolonging sleep duration. The mechanism mainly involves: (1) neurotransmitter regulation, by increasing GABA in brain tissue, reducing the Glu/GABA ratio and regulating the 5-HT level of the hypothalamus; (2) Metabolic pathway intervention—callback of seven differential metabolites, involving glycerophospholipid metabolism, tryptophan metabolism, ascorbic acid metabolism, linoleic acid metabolism, sphingolipid metabolism, purine metabolism, and lysine metabolism; (3) By regulating the intestinal flora, reducing the insomnia-related marker bacteria (*Mycobacterium heterophilum*) and optimizing the proportion of the flora, FM exerts a sedative and hypnotic effect by regulating the hypothalamic metabolic network and neurotransmitter balance. Compared with synthetic sleeping pills, FM can be used as a natural alternative or adjuvant therapy for traditional sleeping products, and has potential advantages in safety and multi-target efficacy. However, it is necessary to verify its effectiveness and optimal dose in the human body through clinical trials. Although these findings highlight the potential of FM as a nutritional supplement for sleep disorders, further verification is needed to confirm its transformation value in the human body.

## Figures and Tables

**Table 1:** Amino acid scale before and after fermentation of *Moringa oleifera* leaves.

Amino Acid Types	UFM (ug/g)	FM (ug/g)
Gly	84.13 ± 2.76	157.51 ± 3.73
Ala	4320.29 ± 32.35	3817.81 ± 104.13
Ser	2184.95 ± 50.05	2734.49 ± 67.93
Pro	1904.77 ± 56.07	1755.85 ± 48.79
Val	2306.58 ± 61.21	1977.63 ± 27.11
Thr	2381.01 ± 62.15	2055.19 ± 40.44
Ile	1566.61 ± 29.95	1419.81 ± 39.63
Leu	2035.86 ± 45.37	1954.83 ± 24.83
Asn	9664.22 ± 81.62	10,652.51 ± 267.69
Orn	2637.48 ± 41.99	22.51 ± 1.30
Asp	1845.35 ± 46.61	1333.30 ± 23.00
Gln	1563.28 ± 19.90	4220.03 ± 55.37
Lys	314.63 ± 4.65	602.95 ± 16.51
Glu	3433.15 ± 65.13	2337.14 ± 85.89
Met	18.48 ± 0.85	78.38 ± 0.84
His	1094.90 ± 14.81	1159.44 ± 19.09
Phe	3524.23 ± 64.26	3616.08 ± 88.18
Arg	24.35 ± 1.48	3315.39 ± 27.02
Tyr	806.17 ± 12.36	899.82 ± 27.88
Trp	1402.16 ± 37.66	1447.53 ± 26.25

**Table 2:** Effects of FM on hypothalamic neurotransmitters in mice (* *p* < 0.05, ** *p* < 0.01).

Groups	5-HT (n/g)	Glu (µg/g)	GABA (µg/g)
Control	183.557 ± 42.512	19.865 ± 7.734	4.702 ± 0.556
FM-L	216.362 ± 53.114	17.956 ± 6.498	4.979 ± 0.726
FM-H	272.322 ± 76.095 *	15.814 ± 2.389 **	6.261 ± 0.766 **
DZP	293.082 ± 35.910 **	13.771 ± 2.274 **	6.568 ± 1.078 **

**Table 3:** Analysis of FM components penetrating the brain.

Name	Formula	HD:MD	Source
3,6,7-Trihydroxy-4′-methoxyflavone 7-rhamnoside	C_22_H_22_O_10_	up	*hrysanthemum morifolium*
(S)C(S)S-S-Methylcysteine sulfoxide	C_4_H_9_NO_3_S	up	*Allium sativum*, *Allium cepa*
Cinncassiol A	C_20_H_30_O_7_	down	*Cinnamomum cassia*
Palmitoyl Ethanolamide	C_18_H_37_NO_2_	down	*Glycine max*, *Helianthus annuus*
Nepetaside	C_16_H_26_O_8_	down	*Nepeta cataria*
8(R)-Hydroperoxylinoleic acid	C_18_H_32_O_4_	down	*Glycine max*, *Helianthus annuus*
Dodecyl Hydrogen Sulfate	C_12_H_26_O_4_S	down	*Gleditsia sinensis*, *Cocos nucifera*
Melleolide	C_23_H_28_O_6_	down	*Armillaria ostoyae*, *Armillaria gallica*
Melatonin	C_13_H_16_N_2_O_2_	down	*Vitis vinifera*, *Olea europaea*
Uric Acid	C_5_H_4_N_4_O_3_	down	*Adenine*, *Guanine*

**Table 4:** Binding energies and residues involved in hydrogen bonding, hydrophobic interactions, and electrostatic interactions of important sleep-aiding compounds and key target proteins.

Complexes	Binding Energy (kcal/mol)	Hydrophobic Interactions	Hydrogen Bonding	Electrostatic
GABA/GBRA1	−4.3	\	Asn414, Asp420, Arg424	Glu277
5-HT/HTR1A	−6.8	Tyr96	Ala93, Gln97, Asp116	\
5-HT/HTR2A	−6.5	Val156, Phe340	Phe234, Val235, Ser239	\
Glu/GRM5	−4.8	\	Lys186, Phe188, Glu458, Asn459	\
5-HT/HTR1B	−6.3	Ile130, Cys133, Ala216, Phe330, Phe331	Thr134	\

## Data Availability

The original contributions presented in this study are included in the article/Supplementary Materials. Further inquiries can be directed to the corresponding author.

## Supplementary Materials

The following supporting information can be downloaded at: https://www.mdpi.com/article/10.3390/foods14172952/s1, Figure S1: GABA standard curve.
