# Supplementary figures and images for: Integrated Approach Reveals Fermented Moringa oleifera Leaves Extracts’ Impact on Mouse Sleep
Source: Foods. 2025 Aug 25;14(17):2952. doi: 10.3390/foods14172952 (PMC12428357; doi:10.3390/foods14172952)

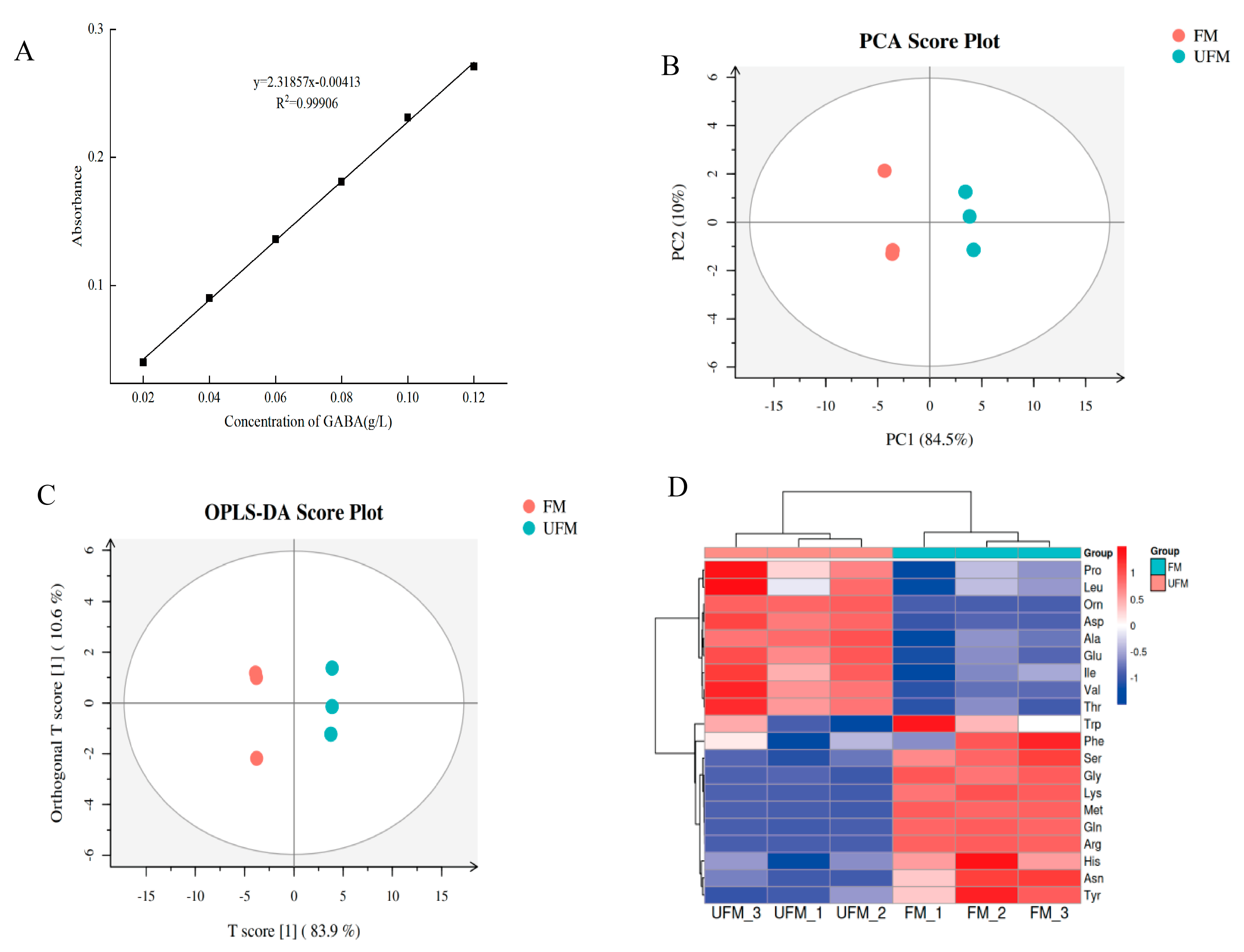

Supplement: Supplementary file 1 [file foods-14-02952-s001.zip › foods-3789761-supplementary.png]
